# Circulating FGF21 vs. Stress Markers in Girls during Childhood and Adolescence, and in Their Caregivers: Intriguing Inter-Relations between Overweight/Obesity, Emotions, Behavior, and the Cared-Caregiver Relationship †

**DOI:** 10.3390/children9060821

**Published:** 2022-06-02

**Authors:** Eirini V. Christaki, Panagiota Pervanidou, Ioannis Papassotiriou, Aimilia Mantzou, Giorgos Giannakakis, Dario Boschiero, George P. Chrousos

**Affiliations:** 1 Childhood Obesity Clinic, First Department of Pediatrics, School of Medicine, National and Kapodistrian University of Athens, “Aghia Sophia” Children’s Hospital, 11527 Athens, Greece; ppervanid@med.uoa.gr (P.P.); amantzou@med.uoa.gr (A.M.); chrousge@med.uoa.gr (G.P.C.); 2 Unit of Developmental and Behavioral Pediatrics, First Department of Pediatrics, School of Medicine, National and Kapodistrian University of Athens, “Aghia Sophia” Children’s Hospital, 11527 Athens, Greece; 3 Department of Clinical Biochemistry, “Aghia Sophia” Children’s Hospital, 11527 Athens, Greece; ipapassotiriou@gmail.com; 4 Computational Biomedicine Laboratory, Institute of Computer Science, Foundation for Research and Technology Hellas (FORTH), 70013 Heraklion, Greece; ggian@ics.forth.gr; 5 Institute of AgriFood and Life Sciences, University Research Centre, Hellenic Mediterranean University, 71410 Heraklion, Greece; 6 BioTekna Co., 30020 Venice, Italy; dario.boschiero@gmail.com; 7 University Research Institute of Maternal and Child Health and Precision Medicine and UNESCO Chair on Adolescent Health Care, 11527 Athens, Greece

**Keywords:** FGF21, stress, salivary cortisol, hair cortisol, childhood obesity, externalizing problems, caregiver stress

## Abstract

Fibroblast growth factor-21 (FGF21) acts on several brain regions, including the hypothalamic paraventricular nucleus, which is involved in the regulation of the hypothalamic-pituitary-adrenal (HPA) axis. The purpose of this study was to investigate the interrelations between FGF21 and stress indices in girls, as well as in their caregivers. 78 girls, aged between 5 and 15 years, were studied; 50 of them were overweight and obese (OB) and 28 in the control group (C). Serum FGF21 and hair and diurnal salivary cortisol were measured. Children participants filled in the Children’s Depression Inventory (CDI) and the State-Trait Anxiety Inventory for Children (STAIC), while their caregivers filled in the State-Trait Anxiety Inventory (STAI), the Perceived Stress Scale (PSS), and the Holmes-Rahe Stress Events Scale (HRSES). The OB group girls had significantly higher levels of FGF21 than the C group (*p* < 0.001). In contrast to the C group, in whom FGF21 levels were positively correlated with both hair and salivary AUCg cortisol concentrations (*p* = 0.045 and *p* = 0.007, respectively), no such correlations were observed in the OB group. In the caregivers of the OB group, STAI-state (r = 0.388, *p* = 0.008), STAI-trait (r = 0.4, *p* = 0.006), PSS (r = 0.388, *p* = 0.008), and HRSES (r = 0.358, *p* = 0.015) scores, all correlated positively with the FGF21 levels of the children under their care. FGF21 concentrations positively correlated with hair and salivary cortisol levels in the C group only. These findings may represent an interesting correlation dictated by bi-directional empathy links between the primary caregivers and the children under their care.

## 1. Introduction

Childhood/adolescence overweight and obesity prevalence has significantly increased worldwide during the last decades. This is probably due to marked lifestyle changes, including a poor diet composition and an increasingly sedentary behavior. There is also growing evidence that the development of obesity may also be the result of chronic stress through multiple pathways, including behavioral changes, specific macronutrient preferences, and stress hormone dysregulation [1,2]. One of the primary physiological responses to stress is the activation and the change in the circadian rhythmicity of the hypothalamic-pituitary-adrenal (HPA) axis [2,3,4,5]. During stress of any etiology, the activation of the hypophysiotropic neurons in the hypothalamic paraventricular nucleus (PVN) promotes the release of adrenocorticotropic hormone (ACTH) into the systemic circulation, which then acts upon the adrenal cortex, stimulating cortisol secretion. 

Fibroblast growth factor-21 (FGF21) is a stress-inducible hormone [5] produced by the liver in response to metabolic stressful stimuli, such as ketosis [4], and crosses the blood-brain barrier to act on several brain regions, including the PVN, which are involved in the regulation of the HPA axis [6]. Exogenous FGF21 administration to mice stimulates glutamatergic neurons in the ventromedial hypothalamus [6] and suppresses sugar intake without increasing energy expenditure, suggesting that this hormone plays an important role in body weight management. Serum levels of FGF21 are elevated in obese humans [7], and this protein has been suggested as a potential biomarker of Metabolic syndrome (Mets) and Type 2 Diabetes Mellitus (T2DM) [8]. In addition, Jelenik et al. (2018) investigated the possible role of FGF21 in the metabolic responses to stress in adults and suggested that this hormone mediates long-term adaptations of tissue insulin sensitivity. As studies on FGF21 in the pediatric population are limited, we examined the possible biological and psychological implications of this hormone in control or increased body weight children and adolescents, as well as the possible interrelations of FGF21 and the HPA axis, as it has been reported that FGF21 stimulates the HPA axis [9,10]. Their parents (and/or caregivers) also had their stress and anxiety levels assessed through specific questionnaires. It has been proposed that parental chronic stress assessed through hair cortisol concentration, alongside with poor socioeconomic status, can affect the levels of hair cortisol concentration of children under their care [11,12].

Based on the above, the following research questions were formulated and investigated in this study:

- Since FGF21 is a stress-inducible hormone, is there a correlation between circulating levels of FGF21 and other stress indices, such as hair or salivary cortisol in girls with increased body weight or in girls in the control group?
- Since FGF21 is a stress-inducible hormone, is there a correlation between serum FGF21 concentrations and anxious or depressive symptoms or behavioral problems in the participants of the study and/or their caregivers?

As there are no previous studies exploring the possible associations of FGF21 with stress and anxiety or depression indices, this was an exploratory study.

## 2. Methods

### 2.1. Participants

A longitudinal lifestyle interventional study was performed in which a cross-sectional analysis of baseline parameters of the participants took place. A total of 78 Greek child/adolescent girls participated in the study, aged 8.75 ± 2.25 years; they were recruited from the Childhood Obesity Clinic of the First Department of Pediatrics, School of Medicine, National and Kapodistrian University of Athens, “Aghia Sophia” Children’s Hospital, Athens, Greece, from March of 2014 to July 2016. Only girls were included in the study so as to avoid gender biases, mostly because differences in FGF21 levels between genders in children and adolescents were reported in a previous study [13]. Baseline evaluation included medical history, assessment of psychosocial and anthropometric parameters, blood testing and Bioelectrical Impedance (BIA) measurements (Biotekna srl. Venice, Italy). The study population was divided into 2 groups: control group C (n = 28) and overweight and obese group OB (n = 50), which included both overweight and obese subjects. The overweight and obese subjects were merged into one group, as there were no statistical differences (Mann-Whitney U test) in most variables investigated in this study (FGF21, insulin, Vitamin D, Tanner stage, Waist-to-Hip ratio, STAIc-state, STAIc-trait, CDI, exercise, family income) between overweight and obese participants. Control group participants were recruited by advertisement, whereas 2 of the sudy participants in the C group were siblings of participants in the OB group. Exclusion criteria were: chronic use of any medication for a pre-existing diagnosis, a mental disorder diagnosis, pre-existing psychopathology, genetic disorders affecting growth and puberty, and pre-existing or concurrent chronic illnesses, such as cardiovascular, rheumatologic, and renal diseases. Primary caregivers were informed about the purpose and the objectives of the research protocol and signed a written informed consent form.

### 2.2. Hair Cortisol Sampling

Scissors were used to obtain hair samples (about 1 cm of hair) with from the posterior vertex, as possible close to the scalp. Paper folders were used to store the samples at room temperature before their analysis. As hair grows about 1 cm per month, the sample obtained can evaluate hair cortisol levels representative of the month prior to sampling [14]. The samples (with a weight of ~20 mg) were placed in grinding tubes (Precellys Lysing Kits, Bertin Technologies, Montigny-le-Bretonneux, France), which led to their lysis and homogenization at 5000 rpm for 7 cycles (1 min each), using a special homogenizer (Minilys, Bertin Technologies) and Precellys lysing kit tubes (tissue grinding CKMix50-R). After this, we added 1 mL of methanol 99.8% and the powder-form hair was extracted at room temperature by shaking for 16 h. The tubes were then centrifuged using the Biofuge 13 (Heraeus Instruments, Hanau, Germany) at 10,000× *g* for 5 min, the extract (700 μL) was transferred to a glass tube and left at room temperature for evaporation of methanol until they were completely dried (about 72 h later). Then, there was sample reconstitution in 100 μL phosphate-buffered saline (with a pH of 8.0, 1xPBS), which was vortexed for about 1.5 min. Before analysis, samples were vortexed again. Finally, samples were analyzed by using an automated Electrochemiluminescence immunoassay (ECLIA) “Cortisol II” using the automated analyzer Cobas e411 (Roche Diagnostics, Basel, Switzerland). The inter-assay precision coefficients of variation for hair cortisol ranged from 3.6% to 11.8% and the lower detection limit was 0.054 ug/dL according to the manufacturer. 

### 2.3. Saliva Sampling

The saliva samples were collected by the participants six times a day using special salivettes (Sarstedt, Hildesheim, Germany) with the help of their caregivers. They were collected throughout one day approximately at 8.00, 8.30, 12.00, 15.00, 18.00 and 21.00, according to written instructions provided to the caregivers. Instructions to abstain from food and drink intake for 30 min prior to the sample collection were included in the leaflet given to the caregivers. Participants needed to chew the tube’s synthetic swab for 1 min, then to store it in the salivette at a temperature of 0–4 °C until their next visit about 3 days later. Then, the samples were centrifuged at 3000 rpm for 5 min and kept at −80 °C, until their time of analysis. Cortisol was measured by the automated analyzer Cobas e411 (Roche Diagnostics) via an electrochemiluminescence immunoassay (ECLIA). The area under the curve (AUC) of the total output of cortisol was estimated with respect to the ground (AUCg) using the concentrations of the six salivary samples collected in one day. There were less than 9% missing samples. In this case, the average of the preceding and following sample values were employed in the analysis. The inter-assay coefficients of variation for salivary cortisol ranged from 3.6% to 11.8% and the lower detection limit was 0.054 ug/dL according to the manufacturer.

### 2.4. Blood Sampling

Blood sampling took place between 8:30 p.m. and 10:00 a.m., after fasting for 12 h. Serum glucose, insulin, triglycerides, total cholesterol (TC), high-density lipoprotein cholesterol (HDL), low-density lipoprotein cholesterol (LDL), vitamin D, aspartate aminotransferase (SGOT), alanine aminotransferase (SGPT), and gamma-glutamyltransferase (γGT) were measured using the Clinical Chemistry analyzer Cobas 6000 (Roche Diagnostics, Basel, Switzerland). Serum cortisol and fasting insulin were measured using the electrochemiluminescence immunoassay principle by the Cobas e411 analyzer (Roche Diagnostics). Serum ferritin levels were assessed by electrochemiluminescence with the Cobas 411 immunoassay analyzer.

Levels of serum FGF-21 levels were determined in all of the patients on admission and also once in controls using an immunoenzymatic technique (Human FGF-21, R&D Systems, Minneapolis, MN, USA). According to the datasheets of the manufacturer, the intra-assay coefficients of variation (CVs) ranged from 5.2% to 10.9% and the inter-assay CVs ranged from 2.9% to 3.5%. We evaluated 55 assays; the minimum detectable dose of human FGF-21 had a range of 1.61–8.69 pg/mL.

### 2.5. Childrens’ Questionnaires

Children and adolescent participants completed the Children’s Depression Inventory and the State-Trait Anxiety Inventory for Children (STAIC-state and STAIC-trait) and tested for their validity and reliability in the Greek population in 2003 [15]. The STAIC inventory consists of 40 self-report questions with a 3-point scale, in which 20 questions focus on the child’s current level of anxiety (state), and 20 questions focus on the child’s general anxiety level (trait). This questionnaire was used to distinguish between a general tendency to anxious behavior as one’s personality internal characteristic, and anxiety, as a fleeting emotional state.

Children’s Depression Inventory (CDI) is a self-reporting test that contributes to the recognition of cognitive, affective and behavioral signs of depression in children and adolescents. The CDI has been tested for its validity and reliability in the Greek population in [16]. The scoring of both questionnaires was used in the statistical analysis.

### 2.6. Caregivers’ Questionnaires

All of the caregivers’ questionnaires were completed by the caregivers regarding their own condition, stress and anxiety levels, except for the child behavior checklist. The caregivers completed the State-Trait Anxiety Inventory (STAI) developed by Spielberger et al. in 1973 [17] and tested for its validity and reliability in the Greek population [18]. The State-Trait Anxiety Inventory is a brief self-rating scale for the assessment of state and trait anxiety. In addition to STAI, caregivers completed the Perceived Stress Scale (PSS), a psychometric tool to assess “perceived stress”, with a higher score indicating greater stress. The questionnaire has previously been validated in the Greek language [19]. Finally, caregivers filled in the Holmes and Rahe stress inventory [20] (HRSES) that consists of 43 stressful life events that took place during the past year of the individual’s life [20] (HRSES), which consists of 43 stressful life events that took place during the last year of the individual’s life. Such events were found frequently to precede illness onsets, thus, potentially affecting the individual’s health. In this test, the participants select the events and a score is calculated by summing up the corresponding weights associated with each selected event. This score indicates the probability that the subject will become ill. A total score of ≤150 is normal, while a score between 150 and 300, implies that there is an almost 50% chance of getting ill in the next 2 years.

The Greek version of the child behavior checklist (CBCL) was completed by the caregivers regarding the children under their care and was used to assess internalizing and externalizing symptoms in children [21]. As conceived by Achenbach in 1991, 26 internalizing symptoms refer to problems of withdrawal, somatic complaints, and anxiety/depression, while externalizing symptoms are manifested as delinquent and aggressive behaviors. The data obtained through this questionnaire were inserted into the ASEBA (Achenbach System of Empirically Based Assessment) computerized system and t-scores and percentiles of symptoms, based on Greek norms, were calculated. This questionnaire was filled-in by the primary caregivers of the girls participating in the study.

### 2.7. Statistical Analysis Methods

All study variables were initially checked for their normal distribution (Kolmogorov-Smirnov test). The statistically significant mean differences between the 2 study groups C and OB were assessed using the Mann-Whitney test at a significance level α = 0.05. 

Possible correlations between the study parameters were assessed using the Spearman correlation test, adjusted for sex and Tanner stage. Categorical data were assessed using the chi-square test. For all the tests the significance level was set at 0.05.

The excessive body weight and fat (including overweight and obesity) prevalence, was defined as the 88th percentile BMI-SDS cut-off points of the International Obesity Task Force (IOTF) criteria [22]. BMI z-scores were calculated based on the contemporary Greek growth charts [23].

To perform the calculations and the statistical analyses, the Statistical Package for the Social Sciences (SPSS, version 21; SPSS Inc., Chicago, IL, USA) and Matrix Laboratory (MATLAB v.2018b) were utilized. 

## 3. Results

Participants’ sociodemographic (age, sex), anthropometric (BMI z-score, Tanner pubertal stage), psychometric and bioimpedance variables were calculated and tested for normality. The overweight and obese participants were grouped together as one due to the absence of statistically significant differences in sociodemographic, anthropometric, developmental and body composition variables. Statistical differences between groups C and OB were assessed using the Mann-Whitney independent samples test and the results are summarized in Table 1. 

There were no significant differences between groups in age, Tanner stage, levels of exercise and screen time. The social/economic status was assessed through parents’ education and family income. There were no differences in the parents’ education and their annual income. The study design ensured that there were no significant differences between the two groups on the above parameters, so as to minimize a bias effect on stress, inflammation, body composition and metabolic biomarkers, or other confounding factors. 

The anthropometric variables (BMI z-score, waist-to-hip (WtH) ratio) and body composition analysis parameters (total body water, fat mass, skeletal muscle mass) were, as expected, significantly different between the 2 groups (controland overweight/obese) (*p* < 0.001). 

### 3.1. Serum FGF21 and Body Composition

**Figure 1:**
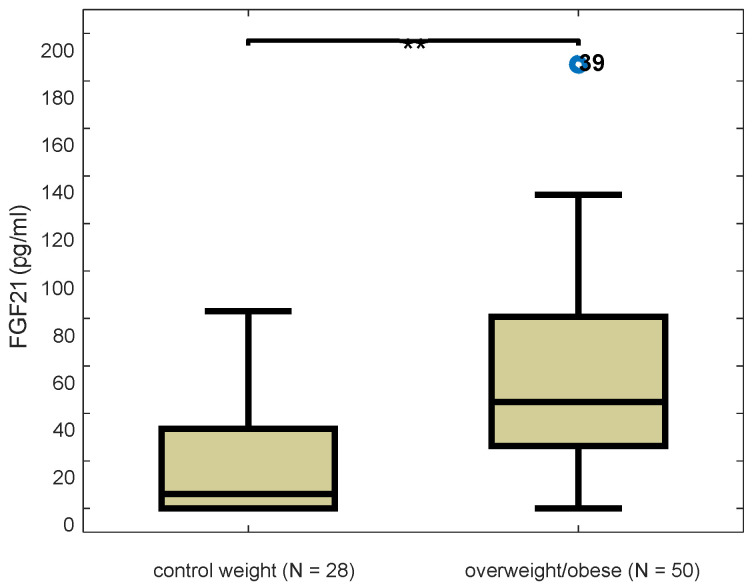
Boxplot distributions of serum FGF21 levels in control and overweight/obese participants (z = −3.725, *p* < 0.001), Mann-Whitney test. Blue dot indicates outlier value and double asterisk ** significance at 0.01 level.

Serum FGF21 levels of overweight/obese girls were significantly higher than those of the control group using the non-parametric Mann-Whitney test (z = −3.725, *p* < 0.001). The boxplot distributions of FGF21 of both groups are presented in Figure 1.

Serum FGF21 concentration was strongly positively correlated with body weight and composition variables, including BMI z-score (r = 0.50, *p* < 0.001), waist to hip ratio (r = 0.41, *p* < 0.001), fat mass percentage (r = 0.49, *p* < 0.0001), and total body water (r = 0.47, *p* < 0.001). 

### 3.2. Serum FGF21 and Psychometric/Behavioral Factors

Levels of circulating FGF21 were investigated regarding potential correlations with psychometric characteristics of both the participants of the study and their primary caregivers. The possible correlations of serum FGF21 and the scoring of participants’ psychometric scales (CDI, STAICstate, STAICtrait) were tested. In the C group there was no correlation between variable pairs. In the OB group, however, FGF21 was significantly correlated with the CDI scale (Spearman r = 0.364, *p* = 0.023).

Regarding the psychometric questionnaires filled out by the caregivers, serum FGF21 did not correlate with any of the scales in the questionnaires of the caregivers of the girls in the C group using the Spearman correlation test. In contrast, serum FGF21 significantly correlated with the STAI-state (r = 0.327, *p* = 0.03), STAI-trait (r = 0.409, *p* = 0.006), and HRSES (r = 0.378, *p* = 0.011) scales in the scoring of the caregivers of the OB group. It is interesting that increased anxiety, depressive symptoms and stress levels of the caregivers, as depicted in all three related questionnaires, had a significant impact on serum FGF21 in children in the OB group only. 

We also examined the correlations of serum FGF21 with behavioral factors using the CBCL questionnaire. Again, no correlation of serum FGF21 with the behavioural and emotional problems assessed by this questionnaire was observed in the C group. However, in the OB group, serum FGF21 was significantly correlated with aggression problems (r = 0.371, *p* = 0.02), externalizing problems (r = 0.436, *p* = 0.005) and oppositional behaviors (r = 0.340, *p* = 0.034), as summarized in Table 2. 

### 3.3. Serum FGF21 and Stress- and Obesity-Related Metabolic Dysregulation Markers

In the OB group, serum FGF21 levels were not correlated with salivary AUCg or hair cortisol concentrations, in contrast to the C group whereby FGF21 levels were positively correlated with both salivary AUCg and hair cortisol concentrations (Pearson r = 0.539, *p* = 0.007 and Pearson r = 0.404, *p* = 0.045, respectively). Interestingly, in the OB group, circulating FGF21 levels were negatively correlated with 25-hydroxy-vitamin D (r = −0.541, *p* = 0.005), controlling for fat mass percentage as a confounding factor; in contrast, such correlation was not observed in the C group.

There were no significant correlations between circulating FGF21 and metabolic biomarkers in the C group, while serum FGF21 was significantly correlated with insulin (r = 0.477, *p* = 0.002), γgt (r = 0.502, *p* < 0.001), FMP (r = 0.025, *p* = 0.025), and HDL (r = −0.294, *p* = 0.05) in the OB group. The results are summarized in Table 3.

## 4. Discussion

Our study evaluated the correlations of serum FGF21 with stress, metabolic, and behavioral indices. Research on FGF21 in children and adolescents is limited, however, as in adults, serum FGF21 has been found elevated in overweight and obese children and adolescents [24]. Circulating FGF21 has been suggested as a potential biomarker for early detection of metabolic syndrome and type 2 diabetes mellitus in adults [8]. In our study, FGF21 levels were significantly higher in the OB than in the C group, while higher levels of FGF21 were associated with an elevated fat mass and WtH ratio. In addition, serum FGF21 levels correlated positively or negatively with a cluster of cardiometabolic risk factors, such as, respectively, insulin and triglycerides vs. HDL and vitamin D, in the OB group, but not in the C group. FGF21 administration in obese mice alleviated hyperinsulinemia and dyslipidemia [25], indicating that the increased serum FGF21 found in our OB group may be a homeostatic compensation to the metabolic stress imposed by body fat accumulation in children and adolescents. However, we should mention that a study in a pediatric age population showed that FGF21 levels had no significant correlation with BMI or body fat percentage in healthy children [13]. 

In the OB group, a significant association between serum FGF21 and CDI scoring was observed, suggesting a coexistence of elevated FGF21 levels and depressive feelings. CDI is one of the most widely used instruments for assessing the presence and severity of depressive symptoms in children and adolescents. It is worth noting that chronic stress has been linked with depressive symptoms [26] and childhood overweight and obesity have been associated with elevated hair and salivary cortisol concentrations, both biomarkers of an activated stress system [27]. It is important to further explore possible mechanisms through which FGF21, which has been suggested as a key regulator in the adaptation to stress in response to diverse stressors, such as nutrient deprivation or overload, exercise, or cold temperature [28] is implicated in the behavioral aspects of the stress reaction and how it acts both in the central nervous system and in the adipose tissue by enhancing insulin sensitivity [29].

Recent evidence in rodents suggested that FGF21 acts via hypophysiotropic neurons in the hypothalamic PVN to activate the HPA axis and to increase corticosterone secretion in vivo [10]. In our study, glucocorticoid secretion expressed as total diurnal salivary cortisol (AUCg), and as hair cortisol concentration, was associated with FGF21 levels only in the C group. To our knowledge, this is the first observation associating directly serum FGF21 and total salivary and hair cortisol levels in children, suggesting that FGF21 plays a role in the physiologic activity of the HPA axis. This correlation was not detected in the OB group, where hair and total diurnal salivary cortisol are normally elevated, implicating different factors in the activation of the HPA axis that has been observed in childhood and adolescent obesity [30]. Excessive body weight in children was associated with elevated hair and salivary cortisol in previous studies [31]. This association may be interfering with a possible association of FGF21 with cortisol. As in the current study FGF21 is elevated in the OB group and hair and salivary cortisol are expected to be influenced by body weight in the OB group, the probable association of FGF21 and cortisol may not be detectable, if the changes are not proportional in parallel.

In our study, serum FGF21 levels were not only correlated with the children’s stress levels, but also with caregiver anxiety and stress (assessed also as the experiencing by them of serious life events), as the scoring of the questionnaires assessing anxiety (STAI) and stress (PSS and HRSES) correlated with the FGF21 levels of the children under their care. It has been suggested that parental stress can affect children’s neurodevelopment and responsiveness to stress even during the prenatal period [32,33], but also later in life during childhood [34,35]. Previous studies have examined the relations between parents’ psychological stress and their children’s stress biomarkers, such as cortisol [36], but this is the first study to examine the possible correlation between parents’ psychological state and FGF21 levels—a stress induced hormone- in their overweight/obese daughters. It has been suggested that parental empathy may shape children’s long term stress reactivity and may affect biobehavioral mechanisms underpinning cross-generation transmission of the stress response [37]. Our findings demonstrate that the parental psychological state may be inter-related with their children’s HPA axis regulation in children and adolescents with excessive body weight. In the latter group, FGF21 levels were also correlated with aggression and externalizing problems and oppositional behavior, as assessed through the CBCL questionnaire filled in by the caregivers. Externalizing problems have been previously linked to increases in HPA axis activity in children and adolescents [38,39], however, FGF21 levels have not been previously associated with such behavioral problems. 

## 5. Limitations of the Study

Overweight and obese subjects were grouped as one because there were no significant differences between the two groups as far as the biomarkers examined were concerned. Another limitation is the fact that in a small number of cases, (less than 9% of the obtained samples), there were missing values of salivary cortisol due to poor compliance of some participants to the diurnal sampling procedure. The AUCg in these cases was computed using the average cortisol concentration of the sample preceding and following the missing sample. Finally, these analyses were cross-sectional and, thus, causality could not be imputed.

## 6. Conclusions

Circulating serum FGF21 levels in overweight and obese girls were higher than those of normal body weight controls. Overweight and obesity were associated with a disturbance of the positive correlation between FGF21 and the stress biomarkers hair and time-integrated salivary cortisol concentrations, which were present in the control group. Similarly, a positive correlation of FGF21 concentrations with the CDI score and serum insulin, and a negative correlation with 25-hydroxy-vitamin D level was observed in the group with excessive body weight, but not in the control group. Increased stress and anxiety levels determined by reliable psychometric instruments, in the caregivers of the OB girls had an intriguing relation to the FGF21 levels of the children under their care. These findings may represent an interesting correlation dictated by bi-directional empathy links between the primary caregivers (mostly parents) and their female children. More and larger studies are needed to support these findings before firm conclusions can be made.

## Figures and Tables

**Table 1:** Participants’ sociodemographic (age, sex), anthropometric (BMI z-score, Tanner pubertal stage), psychometric, body composition and bioimpedance variables, and their statistical significance between groups (C vs. OB).

Parameter	Control (N = 28)	Overweight/ Obese (N = 50)	*p*-Value
**Age**	8.79 ± 1.98	8.67 ± 2.33	0.815
**BMI z-score**	−0.2456 ± 0.537	2.382 ± 1.59	<0.001 *
**Tanner stage**	90% pre-pubertal	80.6% pre-pubertal	0.279
	6.5% mid pubertal	13.9% mid pubertal	
	3.5% post pubertal	5.6% post pubertal	
**Waist-to-Hip ratio**	0.85 ± 0.56	0.91 ± 0.61	<0.001 **
**Levels of exercise (h/week)**	6.23 ± 4.38	5.02 ± 3.19	0.184
**Family income (in income class)**	2.20 ± 0.65	1.81 ± 0.66	0.081
**Parents’ education (years)**	13.90 ± 2.16	15.26 ± 2.5	0.053
**Screen time (h/week)**	12.20 ± 8.15	15.22 ± 9.35	0.166
**STAIC-state scoring**	24.48 ± 3.56	28.34 ± 5.85	**<0.00** 1 **
**STAIC-trait scoring**	31.00 ± 5.06	31.11 ± 6.40	0.866
**CDI scoring**	4.59 ± 3.24	6.25 ± 4.48	0.149
**Total Body Water (% of body weight)**	56.50 ± 4.93	45.74 ± 4.66	<0.001 **
**Fat Mass (% of body weight)**	10.29 ± 6.38	29.72 ± 7.18	<0.001 **
**Fat Mass (Kg)**	3.60 ± 3.17	14.82 ± 8.77	<0.001 **
**Skeletal muscle mass (Kg)**	6.75 ± 2.32	9.27 ± 3.63	<0.001 **
**Skeletal muscle mass (% of body weight)**	23.98 ± 2.85	29.64 ± 3.42	<0.001 **
**Insulin (** **μ** **U/mL)**	6.17 ± 2.62	12.82 ± 8.58	<0.001 **
**Vitamin D (mg/dL)**	27.02 ± 8.74	22.84 ± 8.35	0.149
**Hair cortisol concentration (pg/mg)**	2.90 ± 6.03	2.76 ± 2.54	0.359
**AUCg (μg/dL × minutes)**	5089.99 ± 3057.49	5039.75 ± 4427.02	0.431
**FGF21 (pg/mL)**	19.54 ± 25.80	54.23 ± 42.07	<0.001 **

Note: Bold type and * denotes (*p* < 0.05), ** denotes (*p* < 0.01) statistically significant differences between the 2 groups (control vs. overweight/obese).

**Table 2:** Spearman correlation results between serum FGF21 and behavioral factors in the OB group.

	FGF21 (pg/mL)	
	Correlation Coefficient r	*p*-Value
Social problems	0.311	0.054
Somatic problems	0.263	0.106
Social behaviour	−0.001	0.993
Sluggish cognitive tempo	−0.031	0.852
Somatic problems	0.273	0.093
Anxiety problems	0.234	0.152
Anxious behavior	0.235	0.150
Aggression problems	0.371	0.020 *
Affective behavior	0.228	0.163
Activities problems	−0.064	0.706
Conduct problems	0.299	0.065
Externalizing problems	0.436	0.005 *
Hyperactivity problems	0.281	0.084
Internalizing problems	0.305	0.059
Obsessive behavior	0.121	0.462
Oppositional behavior	0.340	0.034 *
Rule breaking problems	0.266	0.102
School problems	−0.286	0.157
Screen time (h/week)	0.163	0.302
Physical activity (h/week)	0.060	0.719

Bold type and asterisks * denote *p* < 0.05.

**Table 3:** Spearman correlation results between serum FGF21 and obesity-related metabolic dysregulation markers.

	FGF21 (pg/mL)	
	Correlation Coefficient R	*p*-Value
Insulin (μU/mL)	0.477	0.002 **
Fasting glucose (mg/dL)	0.051	0.740
Triglycerides (mg/dL)	0.502	0.000 **
SGPT (U/L)	0.169	0.266
SGOT (U/L)	−0.223	0.142
Fat mass percentage (%)	0.335	0.025 *
Ferritin (μg/L)	0.145	0.346
HDL (mg/dL)	−0.294	0.050 *

Bold type and asterisks * and ** denote *p* < 0.05 and *p* < 0.01, respectively.

## Data Availability

The data presented in this study can be accessed in this URL https://doi.org/10.13140/RG.2.2.33953.33124 (accessed on 16 January 2022).
